# Methods for automating the analysis of live-cell single-molecule FRET data

**DOI:** 10.3389/fcell.2023.1184077

**Published:** 2023-08-15

**Authors:** Jozsef Meszaros, Peter Geggier, Jamie J. Manning, Wesley B. Asher, Jonathan A. Javitch

**Affiliations:** ^1^ Department of Psychiatry, Vagelos College of Physicians and Surgeons, Columbia University, New York, NY, United States; ^2^ Division of Molecular Therapeutics, New York State Psychiatric Institute, New York, NY, United States; ^3^ Department of Molecular Pharmacology and Therapeutics, Vagelos College of Physicians and Surgeons, Columbia University, New York, NY, United States; ^4^ Department of Physiology and Cellular Biophysics, Vagelos College of Physicians and Surgeons, Columbia University, New York, NY, United States

**Keywords:** GPCR, FRET, single-molecule imaging, single-particle tracking, automation, machine vision

## Abstract

Single-molecule FRET (smFRET) is a powerful imaging platform capable of revealing dynamic changes in the conformation and proximity of biological molecules. The expansion of smFRET imaging into living cells creates both numerous new research opportunities and new challenges. Automating dataset curation processes is critical to providing consistent, repeatable analysis in an efficient manner, freeing experimentalists to advance the technical boundaries and throughput of what is possible in imaging living cells. Here, we devise an automated solution to the problem of multiple particles entering a region of interest, an otherwise labor-intensive and subjective process that had been performed manually in our previous work. The resolution of these two issues increases the quantity of FRET data and improves the accuracy with which FRET distributions are generated, increasing knowledge about the biological functions of the molecules under study. Our automated approach is straightforward, interpretable, and requires only localization and intensity values for donor and acceptor channel signals, which we compute through our previously published smCellFRET pipeline. The development of our automated approach is informed by the insights of expert experimentalists with extensive experience inspecting smFRET trajectories (displacement and intensity traces) from live cells. We test our automated approach against our recently published research on the metabotropic glutamate receptor 2 (mGluR2) and reveal substantial similarities, as well as potential shortcomings in the manual curation process that are addressable using the algorithms we developed here.

## 1 Introduction

Over the years, researchers have developed various analysis methods for the automated processing of single-molecule fluorescence resonance energy transfer (smFRET) data to generate insights into the structure and function of mostly purified and reconstituted biomolecules in surface-immobilized preparations (Greenfeld et al., 2012; van de Meent et al., 2014; Juette et al., 2016; Thomsen et al., 2020). At the same time, tremendous strides have been made within the single-particle tracking (SPT) field to develop analysis methods to improve the localization of diffusing single- or multi-color labeled particles within living cells (Jaqaman et al., 2008; Chenouard et al., 2013; Chenouard et al., 2014; Tinevez et al., 2017; McQuin et al., 2018; Roudot et al., 2020; Ershov et al., 2022). While these methods and their applications have been indispensable for analysis of data from smFRET or SPT experiments, there had been no publicly available, automated analysis that combines these methods for tracking smFRET in live cells, likely a contributing factor as to why the use of in-cell smFRET is infrequently reported in the literature. To overcome this challenge of tracking smFRET in live cells, we recently developed an analysis platform, smCellFRET, which generates large numbers of smFRET trajectories and also produces smFRET intensity over time traces for each trajectory as well as smFRET population histograms (Asher et al., 2021).

Using technical advances we developed for smFRET imaging, including the smCellFRET software, we recently successfully carried out smFRET experiments in living Chinese Hamster Ovary (CHO) cells in order to study G protein-coupled receptor (GPCR) dimerization, in addition to structural changes within metabotropic glutamate receptor 2 (mGluR2) (Asher et al., 2021). With this approach, we were able to show agonist-dependent conformational changes within ligand binding domains of mGluR2 dimers as they diffused in the native plasma membrane, which prior to our work had only been studied in the context of detergent-solubilized receptors (Olofsson et al., 2014; Vafabakhsh et al., 2015). Our analysis and technical advances for smFRET data in cells opens up the possibility of studying how the complex cellular environment might influence receptors toward certain conformations or facilitate associations between noncovalently linked receptors such as the Family B secretin receptor (Asher et al., 2021).

**FIGURE 1:**
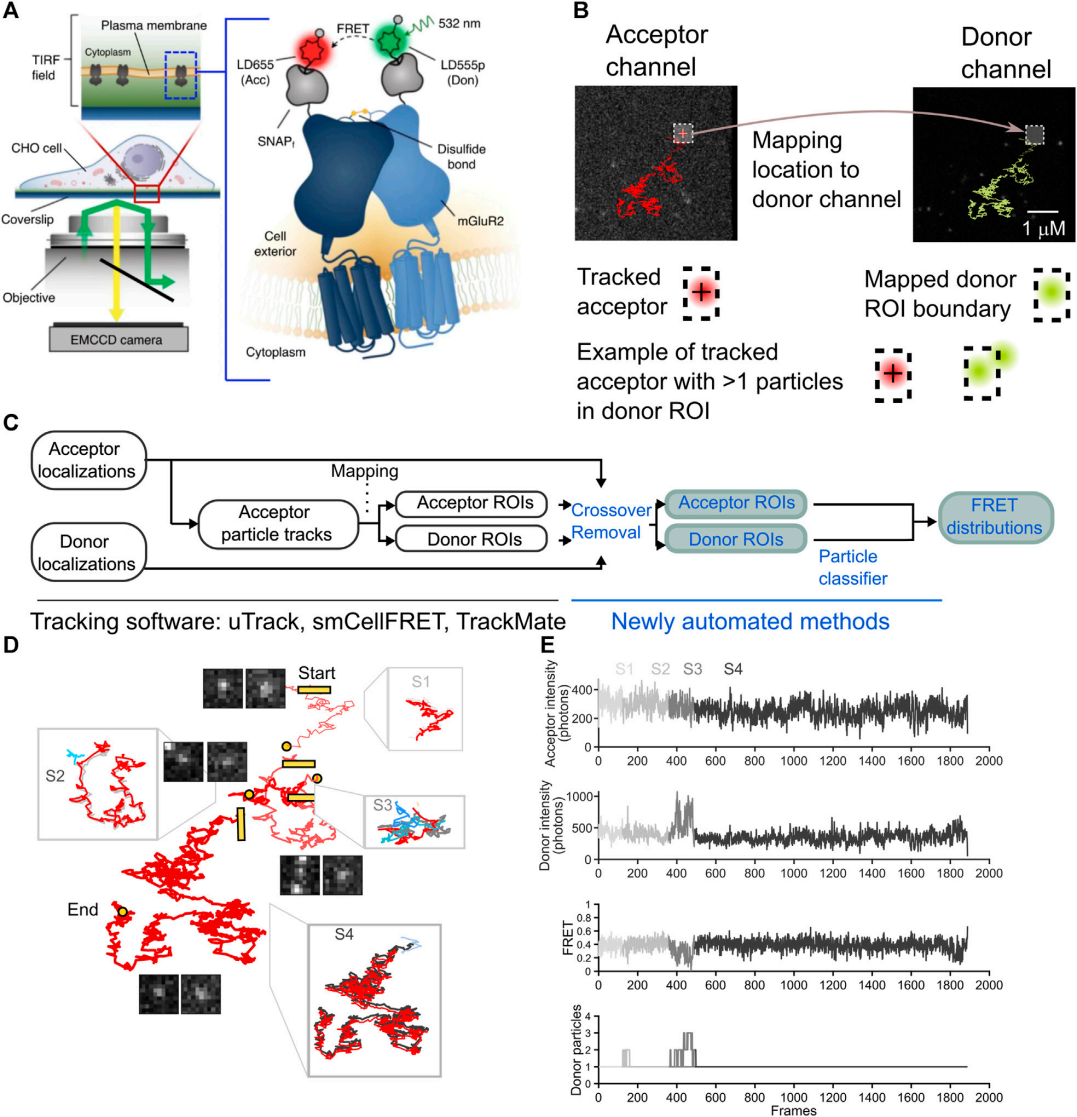
The overview of an smFRET algorithm to remove cross-over in both acceptor ROI and a mapped donor channel ROI. **(A)** Images are collected in the donor and acceptor channel for 4,000 consecutive timepoints at a frequency of 25 Hz (diagram reproduced from Asher et al., (2021). **(B)** A complete smFRET trajectory is shown within the acceptor (red track, left) and donor (green track, right) channels. The sensitized acceptor trajectory is obtained using single-particle tracking and a donor ROI is mapped from the computed acceptor localizations A cartoon diagram beneath the tracks illustrates how an acceptor particle’s track is computed (crosshair, left) and then used to delineate a boundary in the donor channel (hashed box, right) in the bottom row. an illustration of how multiple donors may appear within the boundary and thereby contaminate the estimation of the donor ROI intensity. **(C)** A schematic to describe the steps of the analysis pipeline with the previously published processing steps shown (black text, left) alongside the newly automated steps (blue text, right). **(D)** A representative smFRET trajectory, spanning 1,888 frames from start to end split into segments to illustrate crossing-over events the start of each new segment is labeled with a square and the end of a segment is a circle segments are arbitrarily displaced with respect to each other for ease of visualization for each segment a zoom-in shows the acceptor trajectory’s displacement (gray) along with the most the most likely donor molecule (green) and interfering particles and signals (shades of blue). **(E)** For the smFRET trajectory shown, time traces reveal the progression of the acceptor intensity, donor intensity and FRET, as well as the number of particles within the donor channel search area, as detected by our algorithm on a frame-by-frame basis. the corruption of the intensity and FRET traces is evident in and around frames where the number of donor particles exceeds 1 (segments S2 and S3).

smCellFRET is an open-source software package for use in the MATLAB environment that processes smFRET data of labeled donor and acceptor molecules diffusing in the cell plasma membrane. Data processing in smCellFRET utilizes tracking and motion classification data generated with the SPT software u-track and the DC-MSS analysis method (Jaqaman et al., 2008; Vega et al., 2018; Asher et al., 2021). We used smCellFRET to generate smFRET trajectories, which contain time series data, or time traces, for the location of the acceptor, the inferred location of the donor, and the intensity values computed for each. These smFRET trajectories are computed for FRET events originating from mGluR2 dimers freely diffusing in the cell membrane (Figure 1A). Briefly, smCellFRET will combine outputs from u-track and DC-MSS carried out on image data from sensitized acceptor particles, to obtain tracking and motion state analysis, respectively (Figure 1B). Next, a custom transformation function fit to the optical specifications of the imaging system was used to map acceptor particle locations onto a position in the donor channel. The donor-acceptor trajectory’s twin trajectories are then computed, as the acceptor signal’s location can be used in conjunction with the transformation function to estimate the location and intensity of a region corresponding to the acceptor, only in the donor channel. The intensity values for both the acceptor and donor are computed as the sum of intensities within a 5 × 5 pixel (800 nm × 800 nm) square region of interest centered on the location of the respective particle (Figure 1B). Additionally, when diffusion states are determined for smFRET trajectories, smCellFRET imposes a minimum track length requirement of 20 frames as a requirement of motion state analysis by DC-MSS.

In our early experiments, once we had generated smFRET trajectories, we recognized that multiple particles would occasionally cross into a given donor or acceptor’s path. During such crossing-over events, fluctuations in the donor and acceptor channel ROIs influence the calculation of the FRET value and lead to distortions in the FRET value distributions generated from trajectories where such crossings occurred. To guarantee that crossing-over events were not contributing to the FRET value distributions, a trained specialist would manually inspect both the fluorescence emission in the donor channel and sensitized acceptor emission in the acceptor channel, for every smFRET trajectory used to generate smFRET histograms. This process required watching a video of every smFRET trajectory over its entire trajectory, and we quickly realized that excising individual frames would be unfeasible for hundreds of trajectories per cell, each several hundred frames long. As a compromise, the experimentalist would inspect the video frames of each donor-acceptor trajectory, checking for any crossing-over events, and only preserve those trajectories for further analysis that were entirely free of crossing-over. Given that an experimenter can make a determination at a rate of one frame per second, 8 h is a conservative estimate for the time it would take to hand-curate all of the trajectories from only a single cell. In our previously published dataset, 69% of all traces were rejected, representing 77% of all FRET events. Importantly, much of the excluded data consists of trajectories where interference by other particles happens only in a small subset of frames during that trajectory’s lifetime.

As noted, in our previous work, because of the complexity associated with manually removing these crossings, the entire trajectory would be removed from inclusion in the computation of the FRET distribution, as frame-by-frame analysis and labeling for crossing-over would have been too time-consuming. Background autofluorescence in the cell and heterogeneities across the cell surface made the observation and discrimination of crossing-over events by a human both time-consuming and subjective, leading to potential biases and reduced reproducibility. To overcome these obstacles, we have now developed the necessary analysis for automating the removal of frames with crossing-over events. This allows us to generate an accurate FRET distribution from the data. We additionally provide an intensity-thresholding operation that selects trajectories based on a template-matching approach, using a small subset of trajectories where acceptor photobleaching coincides with a simultaneous rise in donor intensity. The automation we have developed here provides two basic enhancements: (Juette et al., 2016) at the frame level, computing the number of signals detected in the vicinity of each ROI, which could be used to classify an ROI as crowded, and (Thomsen et al., 2020) at the particle level, an unbiased classifier on the intensity levels of an smFRET acceptor and donor to remove those donor-acceptor trajectories where either the acceptor or donor intensities lie outside of a range justified by smFRET principles and our data. These automation steps represent a first foray into uncovering additional, general principles that may be integrated into a more comprehensive, rigorous framework.

## 2 Methods

To develop methods for the automation of crossing-over event removal, we sought to replicate the performance of a manually-curated analysis approach on a dataset obtained using total internal reflection fluorescence microscopy (TIRF) imaging of the SNAPf-tagged membrane-embedded protein, mGluR2, controllably expressed at low levels (Asher et al., 2021). The proteins were stochastically labeled with a mixture of Lumidyne 555p (LD555p) and 655 (LD655), the donor and acceptor fluorophores, respectively (Zheng et al., 2017; Pati et al., 2020). As described in Table 1, data were collected under three conditions of ligand availability (0 µM, 15 µM, and 100 µM glutamate) and processed through smCellFRET. For each cell, smCellFRET generated all of the smFRET trajectories that persisted for 20 frames or more (Table 1, column 2). In each condition, multiple cells expressing mGluR2 covalent dimers stochastically labeled with both acceptor and donor were imaged for 160 s at 25 Hz, which produced mGluR2 FRET events in the acceptor channel. Each smFRET trajectory consists of the number of frames during which the acceptor signal was detectable without interruption by our pipeline (Table 1, column 3). Given that each movie was 4,000 frames, this places an upper limit on the number of frames within a trajectory, which were near 100 frames on average (Table 1, column 4). As a negative control, we also collected data using the single-pass transmembrane domain of the low-density lipoprotein receptor (LDL-TM), which is known to be a monomer that will produce negligible smFRET trajectories (Suzuki et al., 2012; Asher et al., 2021).

**TABLE 1:** The total number of smFRET trajectories extracted from the data collected along with the total number of frames for each trajectory.

	Number of cells	Number of smFRET trajectories	Number of total frames across all trajectories	Average length of a trajectory (frames)
*mGluR2* (Apo)	6	2796	371522	132
+15 μM glutamate	6	2002	201932	100
+100 μM glutamate	6	2841	296079	104
*LDL receptor*	12	71	7770	109

### 2.1 Removal of crossing-over events from smFRET trajectories

For the dataset we used in this study to automate the removal of crossing-over frames, we studied mGluR2 dimers with an acceptor and donor fluorophore on each protomer (Figure 1A). When dimers or long-lived complexes form, these complexes can be tracked for multiple frames (Figure 1B). In our experiments, we track the acceptor particle, and infer the location of the donor from that acceptor’s location at each timepoint (Asher et al., 2021). A region of interest (ROI) centered on the acceptor fluorophore’s point spread function can be used as a bounding box to calculate the total number of photons emitted in either the acceptor or donor channel (Figure 1B). As the sensitized acceptor diffuses within the plasma membrane, the donor and acceptor ROIs will be translated in space from one frame to the next (Figure 1B). Unlike conventional single-particle tracking methods, which are primarily concerned with localizing particles and their motion states, we not only require the localization but must also efficiently collect as many photons emitted as possible from the donor and acceptor particles for generating donor and acceptor intensity time traces with sufficient signal-to-noise. These ROIs are then used to calculate corresponding FRET intensity over time traces that can contain important information related to dynamical changes in structural states. For high FRET states the donor may emit too few photons for detection, which would complicate an alternative approach that seeks to localize the exact donor at all timepoints. Rather, we expect donor photons to be emitted at any pixels within the ROI (Figure 1B). Likewise, we attempt to attain an estimate of the total number of photons emitted by the acceptor. For these reasons, the best approach we have found involves creating ROIs for the acceptor and the donor and computing intensities therein (Figure 1C). With this approach, multiple donors or acceptors may appear within the same ROI, leading to erroneous computations of intensities. To solve this problem, we propose a method whereby all information about donor and acceptor localizations is combined with ROI intensity calculations in order to detect frames in which multiple particles may have contributed to the ROI (Figure 1C).

Due to the nature of stochastically labeled particles, signals in the donor channel can come from receptor dimers labeled with one donor and one acceptor, which represents the population of receptors with smFRET signal, but also from dimers containing two donors or only one donor and no acceptor. Thus, there are far more signals in the donor channel compared to the acceptor channel, which only contains sensitized acceptor particles in an smFRET experiment, leading to relatively uncomplicated tracking in the acceptor channel. Because of the relatively high particle density in the donor channel, there is a potential for overestimating the intensity within the ROI if multiple signals contaminate the donor ROI. Whenever another molecule or aberrant fluorescent signal appears in the proximity of the donor ROI, the computation of the total photons emitted from the original acceptor molecule and donor molecule is compromised. Thus, the accurate calculation of the intensity of the donor and acceptor requires knowledge of every fluorescent signal within a region near the ROI. We calculate the FRET efficiency value, E(t), at each frame in time based on the intensity of the donor and acceptor, with the appropriate corrections α, δ, and γ, for donor-to-acceptor bleed-through, acceptor direct excitation at our imaging wavelength, and the relative detection efficiencies and quantum yields of the fluorophores, respectively (Hellenkamp et al., 2018; Asher et al., 2021):

$$E\left(t\right)=\frac{I_{acceptor}\left(t\right)-\alpha I_{donor}\left(t\right)-\delta I_{total}\left(t\right)}{\gamma {Ι}_{donor}\left(t\right)+\left(I_{acceptor}\left(t\right)-\alpha I_{donor}\left(t\right)-\delta I_{total}\left(t\right)\right)}$$ (1)

In a manual-curation approach, one must verify the integrity of the photon calculation for a given acceptor fluorophore and donor fluorophore by scanning every frame of an smFRET trajectory to determine whether the search area around the ROI has been breached by another molecule or signal (Figure 1B). Multiple particles in the donor channel ROI would diminish the FRET value, while multiple particles in the acceptor channel ROI would increase it (Eq. 1). In our previous work, we would verify for every smFRET trajectory that contributed to the calculation of FRET distribution histograms that donor and acceptor ROIs were uncrossed by any other bright signal throughout the entire trajectory.

Here, we have developed an automated approach that can be easily implemented to compute the total number of potentially interfering signals within a user-defined search area around the ROI. Our algorithm is enabled by the u-track platform’s ability to faithfully provide subpixel localizations for any fluorescent signal in an image frame. We rely on u-track to provide localizations for every such signal in both the donor and acceptor channels. However, in many frames, multiple particles will be present in the vicinity of the donor-acceptor trajectory’s displacement, but not entirely overlap either donor or acceptor so as to disrupt detection. To our benefit, u-track outputs all particle trajectories, regardless of size and even for low intensity particles unlikely to be sensitized acceptors or donors. We therefore leverage this information in order to calculate the density of total particles in the vicinity of the donor-acceptor trajectory (counting the original donor and acceptor, plus any interfering particles). To accomplish this, we first compute two time-varying trajectory-wise distance matrices **D** ^trajectory→acceptor^ and **D** ^trajectory→donor^ which contain, for each frame at all times t when a donor-acceptor trajectory is tracked, the distance between the acceptor in the trajectory and the entire set of signals detected in either the acceptor channel or donor channel (Eq. 2). In our experiments, the donor channel is directly excited whereas the acceptor channel signals are due to sensitized acceptor emission, leading to far more signals in the donor channel, and therefore the number of columns in **D** ^trajectory→acceptor^ will be much less than the number of columns in **D** ^trajectory→donor^.

$$\begin{array}{l} D_{ij}^{pair\to acceptor}\left(t\right)=\sqrt{{\left({\vec{x}}_{i}^{pair}\left(t\right)-{\vec{x}}_{j}^{acceptor}\left(t\right)\right)}^{2}}\\ D_{ij}^{pair\to donor}\left(t\right)=\sqrt{{\left({\vec{x}}_{i}^{pair}\left(t\right)-{\vec{x}}_{j}^{donor}\left(t\right)\right)}^{2}} \end{array}$$ (2)

Once the trajectory-wise distance matrices **D** are computed, a density can be computed *at each timepoint*, ρ_i_(t) for the *i*th donor-acceptor trajectory, as the count of all signals in either the acceptor or donor channel located at a distance less than *d*_min_:

$$\begin{array}{l} \rho_{i}^{acceptor}\left(t\right)=\sum_{j=1}^{N}1\left(D_{ij}^{pair\to acceptor}\left(t\right)<d_{\min}\right)\\ \rho_{i}^{donor}\left(t\right)=\sum_{j=1}^{M}1\left(D_{ij}^{pair\to donor}\left(t\right)<d_{\min}\right) \end{array}$$ (3)

In this equation, the values M and N represent the *total number of located particles* (all localizations detected by u-track) that may cause interference *at a given timepoint t*, in the acceptor or donor channel, respectively. The density function is a sum at each timepoint over all of that timepoint’s potential interfering particles, and the identification function (script 1 in Eq. 3) returns a 1 when the condition inside is met (a particle is localized at a distance < d_min_). We use the notation *pair→acceptor* and *pair→donor* to describe the distance between the acceptor/donor pair under examination and any other particles in the ROI of either the acceptor or donor, respectively. Note that the density must return 1 at a timepoint where the original donor and acceptor of the donor-acceptor trajectory have been successfully located by u-track. At some timepoints, when the smFRET efficiency is very high and approaches 100%, the donor may be nearly completely quenched with little to no detectable fluorescence, leading to no donor track, and ρ^donor^ would be zero for the donor-acceptor at those timepoints. With regard to the choice of d_min_, to reduce the possibility of introducing photons from another signal’s Airy disk, we chose 6 pixels for our value of d_min_, as the ROI we used to calculate intensity around the acceptor and donor is 5 pixels x 5 pixels. In the ideal situation where no crossing-over is ever present in the data, there would be N functions for ρ(t), which would be equal to 1 for all timepoints, and the number of acceptor signals N would be equal to the number of donor signals M. In our data, M >> N and ρ(t) would exceed 1 at many timepoints. For the *i*th donor-acceptor trajectory, we preserve the subset of FRET values where for a given timepoint, both ⍴_i_^acceptor^(t) and ⍴_i_^donor^(t) remained less than or equal to 1 (Eq. 4).

Ei=Eit|ρiacceptort<=1and ρidonort<=1 (4)

This inclusion condition leads to a FRET trajectory where FRET values are discarded when either the acceptor or donor channel contains multiple detected particles at a given t. The remainder of the frames are stitched together to create a subset of the FRET values attained by that acceptor-donor pair. Crossing-over events appear as an smFRET trajectory’s donor or acceptor is crossed by another signal in either the donor or acceptor channel, respectively (Figure 1D). Because FRET is inversely proportional to the donor intensity, the elevation in the donor channel leads to an artifactual diminution in the FRET value at the corresponding timepoint in the trace (Figure 1E).

### 2.2 Eliminating smFRET trajectories with excessive donor intensity or insufficient acceptor intensity

**FIGURE 2:**
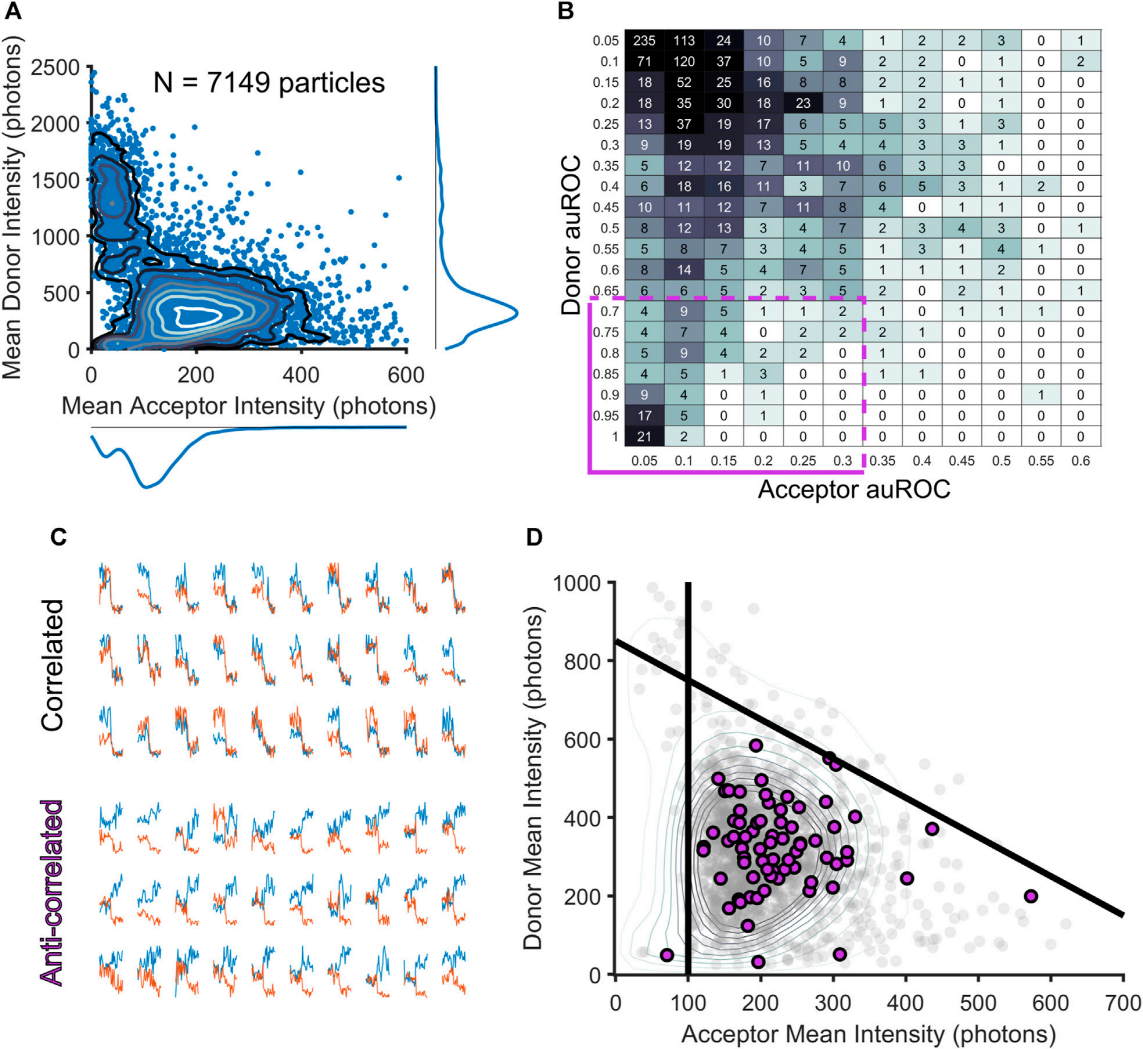
Filtering smFRET trajectories through the assessment of exemplary smFRET donor-acceptor trajectories. **(A)** Scatterplot showing the two-dimensional distribution of mean acceptor and mean donor intensities for each smFRET trajectory, revealing potential clusters of complexes. **(B)** A table of auROC values is computed over all donor and acceptor fluorescence intensity traces to reveal correlation and anti-correlation in the period following acceptor fluorophore bleaching (purple box in the bottom left corner captures trajectories with acceptor bleaching and donor intensity increase). **(C)** Examples of correlated (top) and anti-correlated (bottom) intensity traces. **(D)** Scatterplot with all data in gray with exemplary donor acceptor pairs highlighted in purple. Using these exemplary pairs as a guide to lines can be drawn to select a region containing template smFRET trajectories (purple dots).

The identification of frames with crossing-over events is an important step that provides us with a temporal filter on each smFRET trajectory. As mentioned above, the automated method differs from manual exclusion because it allows for the exclusion of individual frames instead of elimination of entire smFRET trajectories. Thus, many donor-acceptor trajectories would be spared using the automated approach that would have been excluded through manual inspection. To compare the two approaches at the level of donor and acceptor intensities, we created a reduced dimensionality representation of the entire dataset of acceptor and donor trajectories (Figure 2A). To do this, we first used our algorithm to exclude frames in the donor and acceptor channel with crossing-over, then for the remaining frames, we computed an *adjusted mean intensity value* for both the donor and acceptor ROIs throughout a trajectory. The adjusted mean intensity value captures the value of the mean photon count within an ROI once cross-over frames have been removed. We plotted these adjusted mean values against each other in intensity space to visualize the populations of donor-acceptor trajectories that produced our entire dataset (Figure 2A). To guarantee that this reduced representation accurately captures information about each of the donors and acceptors, we used Divisive Segmentation and Clustering (DISC), a MATLAB package, to first idealize the intensity traces of all donors and acceptors and calculated the most likely value of the donor or acceptor intensity (White et al., 2020). Idealization maps noisy time traces to continuous sequences of discrete states, allowing for the computation of dwell times within a given state, and the assessment of a mode intensity (the most highly occupied state for a given trace). For each donor and each acceptor, the mode of the intensity was compared against the mean, to check for correspondence on a per-particle basis. In our dataset, the mode and the means corresponded with R > 0.9 for both the donor and acceptor (Supplemental Figure S1) indicating that the mean of the donor and acceptor is a valid approximation of each particle’s most likely contribution to the overall intensity distributions. This two-dimensional visualization reduces each donor and acceptor to its mean intensity over its entire trajectory. In this reduced representation, it becomes possible to localize populations of acceptors and donors based on their intensity, identify high or unresolvable, low intensity particles, and compare the results from our automated algorithm to the manual approach.

Using this reduced representation of the data, we were surprised to find that the specialist’s manual-selection process not only removed many of the donor-acceptor trajectories with crossing-over that our algorithm detected, but that it also removed nearly all trajectories with high-intensity donor particles, as well as many with donors and acceptors of very low intensities. Thus, the specialist’s process, though it was focused exclusively on removing donor-acceptor trajectories with crossing-over events, effectively served as a highly efficient and specific intensity filter. High levels of fluorescence can be detected in the donor channel when two donor particles colocalize, which would be detected as one particle in many frames using our automated approach, and a specialist would rightly exclude the entire trajectory if the donors revealed themselves as multiple colocalizing particles. For low intensity particles, a specialist may interpret fluctuations in the background of the image as crossing-over events, leading to exclusion of those particles. Thus, the specialist’s exclusion of trajectories with perceived crossing-over, while a limitation in one respect, was also a strength in that it rightly discarded particles with intensity values either too dim to be accurately measured or too bright to be generated by a single donor molecule. In contrast, the algorithm developed here, in focusing entirely on crossing-over, placed these spurious signals back into consideration.

To remedy this undesired consequence of performing crossing-over exclusion at the frame, and not donor-acceptor trajectory level as we had previously done, we devised an additional method to remove aberrant donor-acceptor trajectories from our analysis based on intensity. To do this, we considered that in an smFRET experiment, whenever a tracked acceptor molecule bleaches in one step, it reveals a reliable estimate of the donor and acceptor intensities in a *bona fide* smFRET donor-acceptor trajectory. The donor molecule is released from FRET energy transfer and attains its intrinsic fluorescence intensity, an upper limit set by the photophysics of the fluorescent dye and the imaging conditions. This anti-correlated intensity shift at the time of acceptor bleaching is widely understood to be a reliable signature of smFRET (Juette et al., 2016; Thomsen et al., 2020). However, such donor-acceptor trajectories were rare in our dataset, as this would only occur when donor-acceptor trajectory tracking lifetimes were longer than bleaching lifetimes. In our data, the opposite was true as tracking was typically lost prior to photobleaching (Asher et al., 2021). Thus, only rarely will we capture frames for an acceptor that bleaches.

While photobleaching events are indeed rare, we have found that the number of acceptors present in our experiments provide an adequate sample to use as templates for delineating the boundaries of single-molecule donor and acceptor intensities. Such events are a clear indicator that an observed sensitized acceptor particle occurs via FRET. We thus take advantage of the acceptor molecules that show photobleaching in order to assess the most likely upper and lower limits on the acceptor and donor intensity values possible within our data. Because an smFRET trajectory is defined by the initial appearance of an acceptor particle, the ability to resolve an acceptor as distinct from noise necessitates the choice of a lower limit on the acceptor intensity. We thus took a template-matching approach and extracted trajectories for which we have very high confidence that smFRET was observed. These crossing-over free trajectories that showed anti-correlated bleaching a small subset of the 7,149 total donor-acceptor trajectories (Figure 2A). To extract trajectories that showed an anti-correlated bleaching response, we started by first selecting only trajectories free of crossing-over events (∼10% of all trajectories). For these trajectories, we next computed an area under the receiver operating characteristic (auROC) values as has previously been done in other contexts for reliably and efficiently measuring step-like features in time series data (Stephenson-Jones et al., 2016; Bloem et al., 2017; Li et al., 2017). For each donor and acceptor intensity trace, an auROC compares a window of donor or acceptor intensity frames prior to acceptor bleaching and a window of donor or acceptor intensity frames after acceptor bleaching. The auROC value tends toward 0 in the case where a particle’s intensity level post-bleaching is below the pre-bleaching level at a majority of frames within a window following the bleaching-event; on the other hand, the auROC value will tend toward 1 when more of the intensity values after bleaching are *greater than* the intensity values prior to bleaching. Anything less than a complete step in the intensity profile of a particle after acceptor bleaching will tend to draw auROC values near 0.5. Thus, to discover donor-acceptor trajectories showing anti-correlated bleaching, an acceptor’s auROC value should be close to 0 and a donor’s auROC value should be close to 1. The results of this analysis can be easily confirmed visually to separate correlated and anti-correlated trajectories (Figure 2C). While the smFRET donor-acceptor trajectories that show anti-correlated bleaching consistent with the well-accepted signature of smFRET (Figure 2B, cluster of data in the bottom-left corner) are rare, can be used as a reliable reporter of the most likely values for the minimum acceptor fluorescence and the maximum donor fluorescence (Figure 2D, lower panel). These donor-acceptor trajectories define a tight cluster of acceptor and donor mean intensity values (Figure 2D). The donor maximum threshold is set as the maximum intensity value attained in the window of frames following acceptor bleaching for this smFRET population. The acceptor minimum is set as the lowest-value mean intensity of the smFRET population.

We examined the output of our algorithm capable of combining smFRET trajectory locations with all of the signals detected in either the donor or acceptor channel by u-track. The results of this analysis gave us an insight into where the most crossing-over contamination occurs, either the donor or acceptor channel, and whether such crossing-over leads to tracking loss in the acceptor channel. First, we quantified the number of frames in which multiple particles appear in either the donor or acceptor channel and found that crossing-over events are three times as likely in the donor channel than the acceptor channel (Table 2). This is expected for an smFRET experiment, as the donors, unlike the acceptors, are directly excited by laser light, whereas acceptors must be sensitized by donors to emit photons. Thus, donor signals are present throughout the imaging session and are in far greater abundance. Thus, the greatest contributor to crossing-over errors originates from within the donor channel, creating a potentially significant source of noise that would bias FRET calculations toward lower values. Notably, of the acceptor channel frames containing crossing-over events, only 722 acceptors had any within five frames of the trajectory’s termination, which represents only 16% of the 7,149 donor-acceptor trajectories that we assessed, suggesting that crossing-over in the acceptor channel typically did not lead to the termination of a trajectory.

**TABLE 2:** A summary of counts for the donor-acceptor trajectories analyzed and the frames which contained crossing-over events.

Total number of donor-acceptor trajectories (prior to thresholding)	7,149
Total frames for analysis	519,635
Total frames with more than one particle in search region (donor channel)	100,862
Total frames with more than one particle in search region (acceptor channel)	30,565
Total number of donor-acceptor trajectories where acceptor has more than one particle in search region immediately prior to the end of the acceptor trajectory	722

**FIGURE 3:**
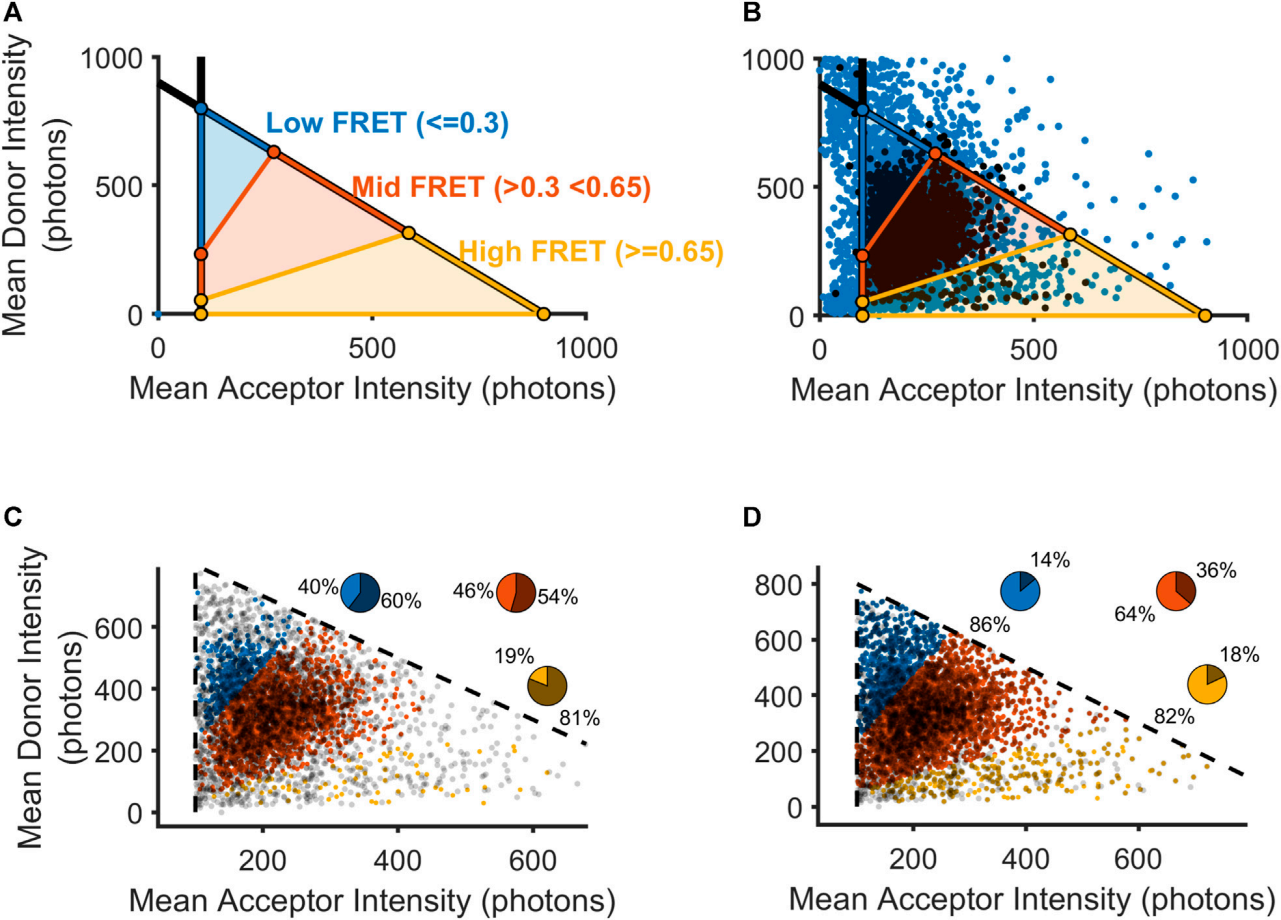
Selection of mGluR2 donor-acceptor pairs using automatically calculated thresholds. **(A)** Visualization of FRET values as they would be calculated within intensity space (donor intensity versus acceptor intensity). Black lines indicate boundaries for the lower bound on the acceptor intensity and the upper bound on the sum of the donor and acceptor intensities. **(B)** Scatterplot showing all donor-acceptor pairs within intensity space (blue) overlaid with those donor-acceptor pairs that passed an experimentalist’s hand-selection criteria (black). **(C)** Scatterplot showing all manually accepted donor-acceptor pairs within intensity space partitioned into three FRET regions. Pie chart insets show the percentage of donor-acceptor pairs that were retained(lighter intensity slice) and those donor-acceptor pairs that were excluded (darker slice). **(D)** Scatterplot showing all-hand selected donor-acceptor pairs as in **(C)**, as well as hand-excluded donor-acceptor pairs that contained non-eclipsing crossing-over events.

As described above, our previous approach focused on removing trajectories where a specialist perceived any crossing-over to be visible in either the donor or acceptor channel, which ostensibly included nearly all of the trajectories with very high donor intensity or very low acceptor intensity (Figure 2A). Without this aspect of hand-selection to narrow our set of donor-acceptor particles with realistic intensity values, we used the adjusted mean intensity values for each donor and acceptor to classify a donor-acceptor trajectory as real signal or noise. As described above, the choice of intensity thresholds was guided by our method of inference based on the intensity values possible for donor-acceptor trajectories showing anti-correlated photobleaching (Figure 2C). To visualize how the operation of either a method of automated thresholding or hand-curation could impact our downstream calculation of FRET distributions, we plotted each donor-acceptor trajectory in intensity space (Figure 3A). We used the acceptor minimum we obtained to place boundaries on the overall distribution of smFRET trajectories. A vertical boundary delineates the ability to resolve an acceptor molecule from noise. Additionally, we computed a donor maximum value. Moreover, we reasoned that the total intensity of the donor-acceptor trajectory should be limited to the sum of the donor maximum value and the acceptor minimum. To denote this maximum total intensity, a diagonal boundary is placed to constrain the sum of the donor and acceptor (total intensity) to an amount equivalent to the sum of the maximum donor mean intensity and the minimum acceptor mean intensity. To calculate the boundaries of the high, intermediate, and low FRET states (Figure 3A), we solved three systems of linear equalities for the respective boundary lines (Eq. 5):

$$\begin{array}{l} E_{FRET}\approx \frac{I_{acceptor}}{I_{acceptor}+I_{donor}}\\ \left\{\begin{array}{l} \frac{I_{acceptor}}{I_{acceptor}+I_{donor}}>0.7\\ I_{acceptor}+I_{donor}<I_{total,\max}\\ I_{acceptor}>I_{acceptor,min} \end{array}\right.\\ \left\{\begin{array}{l} 0.3<\frac{I_{acceptor}}{I_{acceptor}+I_{donor}}<0.7\\ I_{acceptor}+I_{donor}<I_{total,\max}\\ I_{acceptor}>I_{acceptor,min} \end{array}\right.\\ \left\{\begin{array}{l} \frac{I_{acceptor}+I_{donor}}{I_{donor}}<=0.3\\ I_{acceptor}+I_{donor}<I_{total,\max}\\ I_{acceptor}>I_{acceptor,min} \end{array}\right. \end{array}$$ (5)

For visualization purposes, so that we may approximate the boundaries of FRET domains in this intensity space representation, we estimated the FRET efficiency as the theoretical FRET equation, without corrections. The three bracketed systems of equations allow us to place boundary lines around (in order from first to last) the high FRET, medium FRET, and low FRET regions. In our analysis, we accepted for analysis those donor-acceptor trajectories with acceptor and donor intensities falling within one of the three regions delimited by our inferred I_acceptor,min_ and I_total,max_. Once we set discriminative boundaries for the inclusion of viable donor-acceptor trajectories according to the solutions (Juette et al., 2016), we used our intensity space representation to examine where hand-selection produced the exclusion of the greatest number of FRET values. For all donor-acceptor trajectories, we excised any timepoints with detected crossing-over in either the acceptor or donor channel and computed the time-averaged intensity value. During the manual inspection process, a trained specialist was tasked with excluding any donor-acceptor trajectories if they perceived crossing-over events at any time during the trajectory. Based on a visual appraisal of the results of hand-selection in intensity space, we observed that donor-acceptor trajectories containing high-FRET data may have been disproportionately removed for crossing-over events (Figure 3B). Thus, we examined how the specialist’s hand-selected set could have been enlarged if such donor-acceptor trajectories had been reintegrated following our crossing-over detection removal (Figures 3C, D). Indeed, we saw that by reintegrating trajectories with cross-over, we could increase the proportion of donor-acceptor trajectories selected in all clusters, with the greatest increase in the high FRET cluster (bottom, yellow), which had only 19% of the donor-acceptor trajectories included in the hand-selected set to 88% in the adjusted set (Figures 3C, D). The remainder of the trajectories that were excluded by hand curation may have been removed due to human bias or reasons discussed above. When applying our approach, we would include these trajectories as well, given the lack of any objective criteria for excluding them. In our subsequent analysis, we thus relied on the full set of automatically selected trajectories that met our intensity-based criteria, with the removal of frames where crossing-over events were detected by our algorithm.

## 3 Results

**FIGURE 4:**
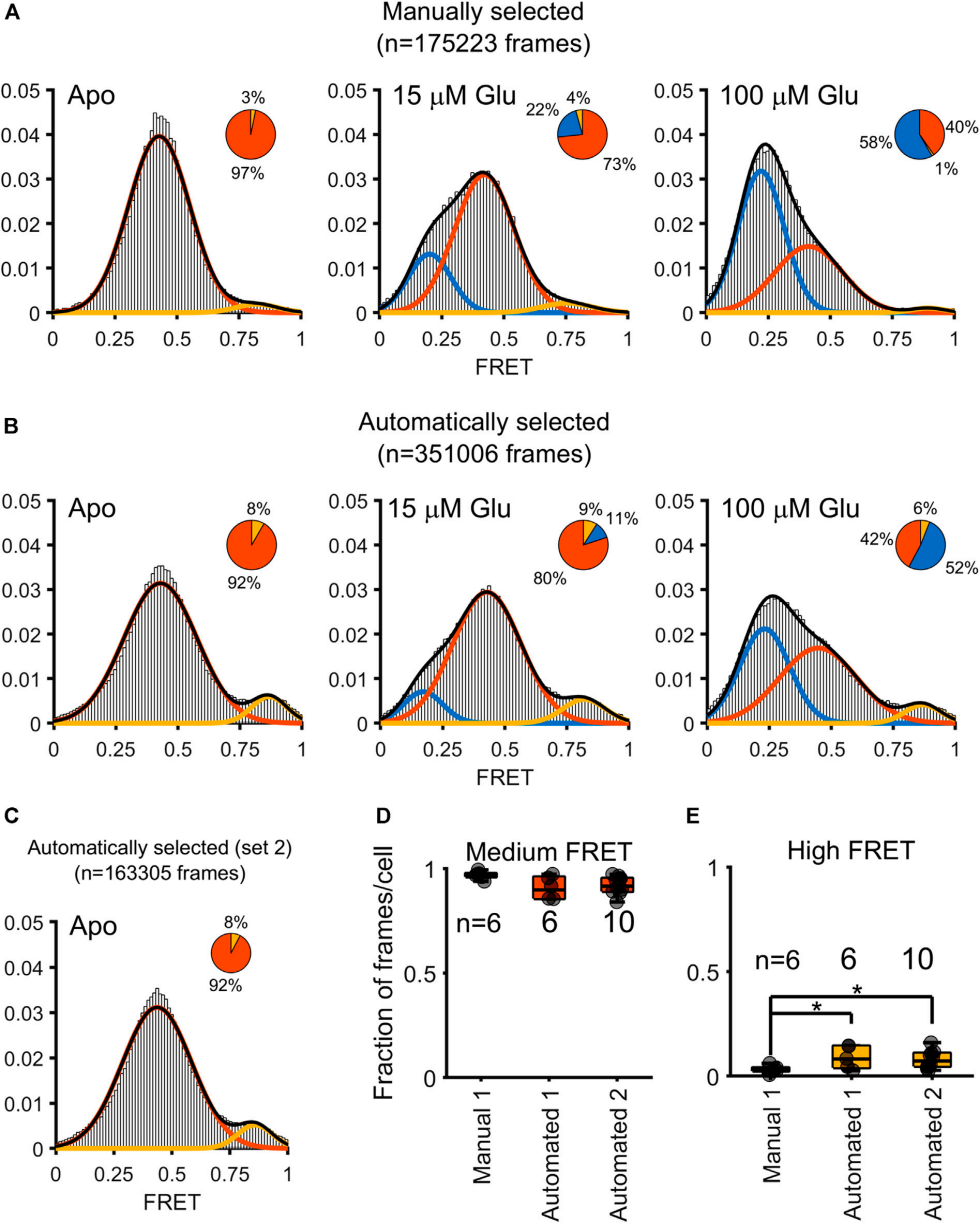
Comparison of FRET distributions obtained from a manually assisted analysis and the automated approach. Gaussian fits to FRET values obtained under three conditions: 0 µM (Apo), 15 µm, and 100 µM glutamate. Three states are obtained (low FRET blue; medium FRET red; high FRET yellow). The pie charts over each histogram reflect the proportion of frames clustered into each state as indicated by the corresponding color. **(A)** Histograms overlaid with Gaussian fits for the FRET values obtained through hand-curation of the data, with pie charts for visualizing the contribution of each state to the histogram. **(B)** Histogram and pie charts to summarize the distribution of FRET values obtained through automatic extraction of FRET values. **(C)** A histogram computed (only Apo state) from ten additional cells. **(D)** Boxplot to depict the contribution of individual cells to the proportion of medium FRET values over the various approaches and datasets, for either the original dataset from Asher, 2020 (Manual 1 and Automated 1) or the automated analysis of the newly-added dataset (Automated 2). Asterisks indicate *p* < 0.05 (independent samples t-test). No significant differences are measured for the proportion of medium FRET state values across the three approaches. **(E)** Boxplot showing an enhanced proportion of high FRET state is measured when using an automated, but not the manual, approach to analysis (independent samples t-test).

Starting with this full set of automatically selected trajectories that met our intensity-based thresholding criteria, with the removal of frames where crossing-over events were detected by our algorithm, we computed the total FRET distributions for all donor-acceptor trajectories within frames with at most one acceptor and one donor present (Figure 4). The results were extracted from tracking *N* = 6 cells for each of the three conditions (Apo, 15 μM, and 100 μM glutamate) (Asher et al., 2021). In comparing the automatically selected FRET dataset to the manually selected set, we notice a doubling in the number of frames (175,223 frames versus 351,006 frames, title of panel A and B), leading to a higher density of data to bolster the overall FRET state distribution. Interestingly, we see the emergence of a high FRET state (∼0.8) within the frames where selection was automated, which is largely obscured in the hand-selected dataset. In that dataset, however, as we previously reported, high-FRET was in fact observed in a small fraction of FRET traces where donor and acceptor showed anticorrelation (Asher et al., 2021). Notably this high FRET state was also observed in smFRET studies with an engineered mGluR2 extracellular domain dimer (Olofsson et al., 2014). To ensure the reproducibility of our method, we repeated all the steps described above on an additional dataset of *N* = 10 cells imaged in the Apo condition (referred to as “Automated 2” in Figures 4D, E). Remarkably, we found near perfect agreement between the FRET distributions in the two Apo condition data sets (Figure 4C). A comparison of the proportion of frames in the intermediate FRET state showed no significant differences between the cells from either the manually selected original set (Manual 1, Figure 4D), the automated original set (Automated 1), or the automated replication set (Automated 1, Figure 4D). When examining the high FRET state, however, the proportion of frames per cell that the manually selected approach clustered were significantly less than the proportion of frames per cell than that in either the analysis of Automated 1 or Automated 2 (Figure 4E). Speaking to the reproducibility of this method, the results from Automated 1 and Automated 2 were not significantly different for either the intermediate FRET state or the high FRET state (Figure 4).

We also applied our new method to a negative control dataset acquired in the same experiment. For the Low Density Lipoprotein transmembrane domain (LDL-TM), a known monomer (Suzuki et al., 2012), our algorithm extracted fewer than 100 frames of smFRET over 6 cells, effectively no FRET events when compared to the mGluR2 data. This result is expected from previous studies (Doumazane et al., 2013; Olofsson et al., 2014; Vafabakhsh et al., 2015) and confirms the ability of our automated method to minimize false positive detections of smFRET.

## 4 Discussion

In our previous work tracking smFRET in live cells, we optimized smFRET particle tracking by expressing low levels of labeled molecules, tracking sensitized acceptor molecules and using these locations to create ROIs for the acceptor channel, as well as for the donor channel via a mapping function, and tuning u-track to provide highly reliable tracking at subpixel resolution (Asher et al., 2021). Nevertheless, we occasionally saw multiple particles within the acceptor ROI, or more often, the donor ROI. In this work, we have primarily concerned ourselves with non-colocalizing crossing-over events, where the point spread function of a nearby donor can dramatically reduce the computed FRET value. In arriving at an automated solution to this problem, we showed that a reduced dimensionality representation of all particles in intensity space could be a useful approach for comparing the results of automation with manual selection. With the aid of the intensity space representation, we discovered that our previous manual selection process that excluded particles containing crossing-over frames also beneficially filtered out spurious low and high-intensity donor-acceptor trajectories. This surprising result required us to develop an additional automated step to filter the output of our smCellFRET pipeline using a template-based approach based on principles of smFRET.

Our intensity thresholding approach here does not rely on visual inspection of images or intensity histograms over the dataset but rather a template-matching approach. We use smFRET donor-acceptor trajectories with a known signature of FRET, anti-correlated bleaching, to justify the selection of a lower boundary on the acceptor and the upper boundary on the donor. It is clear that this logic will apply best in experimental systems where a large amount of data is collected for a given fluorophore, where one donor is expected to meet one acceptor, and where at least some acceptor photobleaching is observed. In some future smFRET experiments, photobleaching may not be observed or expected, in which case another basis for discriminating reliable trajectories would need to be used. For example, a trajectory may reveal a high level of anti-correlation, increasing our confidence that the trajectory is generated by a donor-acceptor pair undergoing smFRET. Such anti-correlated trajectories could be collected to create templates, as we have done here, for estimating the range of intensity values for single acceptor and donor molecules. Alternatively, given a large enough dataset, a spectral clustering approach on the intensity space values could be attempted for sequestering the cloud of template trajectories from background noise. In either case, it is crucial to examine the underlying assumptions of any clustering approach and ensure their validity for the system under investigation.

Our automated approach remedies the manual trajectory selection process’ bias away from high FRET values, introduced by the complete exclusion of donor-acceptor trajectories with any crossing-over frames required by the hand-selection process. By mapping our data in intensity space onto FRET space, we observe that trajectories with high acceptor fluorescence, and therefore higher FRET, invariably persisted for more frames in our experimental pipeline, because we tracked sensitized acceptors and only infer donor locations. The higher intensity of an acceptor leads to tracking over a longer time frame, leading to more opportunities for a manual observer to perceive a crossing-over event. This slight bias toward shorter trajectories and therefore away from high acceptor particles diminished the observation of the high FRET state for mGluR2. The finding of an enhanced proportion of high FRET values is reflected in our previous publication (Asher et al., 2021), where we also observed a high FRET state that was most prevalent in the highest quality traces in which we observed anticorrelated changes in fluorescence. Here our automated methods indicate that this state is more common than we previously estimated.

Ultimately, the enhancements we pursued here led to a nearly two-fold increase in the total data spared after automated curation. While it is possible that by reintroducing donor-acceptor trajectories and smFRET traces that had been rejected by a specialist, we have injected some other type of error. For instance, the minority fraction of trajectories that were rejected by the specialist but contained no detectable crossing-over events may reflect a lack of detection by u-track based on the settings we used. These crossing-over events may have been due to particles that were too dim or not well-fit to a Gaussian and therefore were not tracked by the software. Were it important to consider these low-intensity interferences, one could turn to intermediate data structures within the u-track pipeline, which contain all the local maxima present in an image even before fitting with a Gaussian kernel and could be the basis for a more conservative detector of crossing-overs. Here, we chose to only focus on those tracks which u-track accepted as sufficiently Gaussian and having an adequate signal-to-noise ratio. A track originating from a spot that does not meet these basic requirements would not have sufficient intensity to materially alter FRET, unless the donor and acceptor both had low intensities themselves, a situation that we mitigate against using our thresholding step. For the tracks in the donor and acceptor channels that are identified as potentially interfering with our smFRET donor-acceptor trajectories, our automated approach outputs all the track identities and intensity information. This could enable future studies in which interactions between smFRET donor-acceptor trajectories are studied, or the density of donor-acceptor trajectories in defined membrane areas are related to the FRET values computed. Indeed, our crossing-over detection algorithm can easily be used as a method for obtaining the local density of smFRET donor-acceptor trajectories along an individual trajectory’s trajectory.

We showed that the results of our analysis could be replicated in a different dataset collected at a different time from a different set of cells. Namely, the presence of a high-FRET state we detected through automated analysis of our original, published work can be recaptured in this distinct dataset, at remarkably similar proportions. This bolsters the claim that the glutamate receptor, in living cells, visits at least two distinct conformations even in a condition with no ligand added. The persistence of this state even in increasing levels of ligand provides an interesting insight into the nature of this conformation.

Through our intensity space representations, we sought to develop an intuitive approach for scrutinizing donor-acceptor trajectories and viewing results of selection routines imposed on them. This representation of each donor-acceptor trajectory in intensity space allows us to infer reasonable values for thresholds from mapping template particles, those which had the signature FRET anti-correlated bleaching behavior, onto the overall distribution of particles. Looking within intensity space also provides hints as to where hand-curation may have introduced biases. Moreover, it could help tune tracking, as the donor-acceptor trajectory in the intensity space could be color-coded by its lifetime, revealing potential variability due to particle intensity. Further implementations of this view could be used to examine the diffusion states of molecules spanning intensity space, which would allow probing of the relationship between FRET and diffusion. In another embodiment, points in the intensity space could be color-coded based on their proximity to a biologically relevant landmark in the cell. These relationships could motivate various discriminative classifiers that would give experimentalists novel insights into decoding the relationships between FRET and the cellular milieu in which molecules diffuse.

Ultimately, we believe the optimal approach to scrutinizing smFRET values will integrate a small amount of manual inspection by a trained specialist, especially for populations of donor-acceptor trajectories that are dissimilar from their neighbors in terms of track length, mobility, signal-to-noise, or other features accessible through upstream tracking software such as u-track. The insights of such a specialist can be used to guide and coordinate the automated approaches that can be developed by a data analyst working in conjunction with the experimental team. As imaging experiments become increasingly ambitious and more particle features are used to analyze tracking accuracy and biological variables of interest (Chenouard et al., 2014), the amount of data required will scale exponentially with the dimensionality. By automating aspects of the data curation through meticulous inspection of the various aspects of the donor-acceptor trajectory and the features it generates, the field will increase the throughput of individual experiments and improve the unbiased estimation of particle features as well as the inference of structural and pharmacological parameters of interest.

## Data Availability

The data analyzed in this study is subject to the following licenses/restrictions: Data and code will be made available upon request. Requests to access these datasets should be directed to jm3648@columbia.edu.
