# Supplementary figures and images for: Methods for automating the analysis of live-cell single-molecule FRET data
Source: Front Cell Dev Biol. 2023 Aug 15;11:1184077. doi: 10.3389/fcell.2023.1184077 (PMC10466402; doi:10.3389/fcell.2023.1184077)

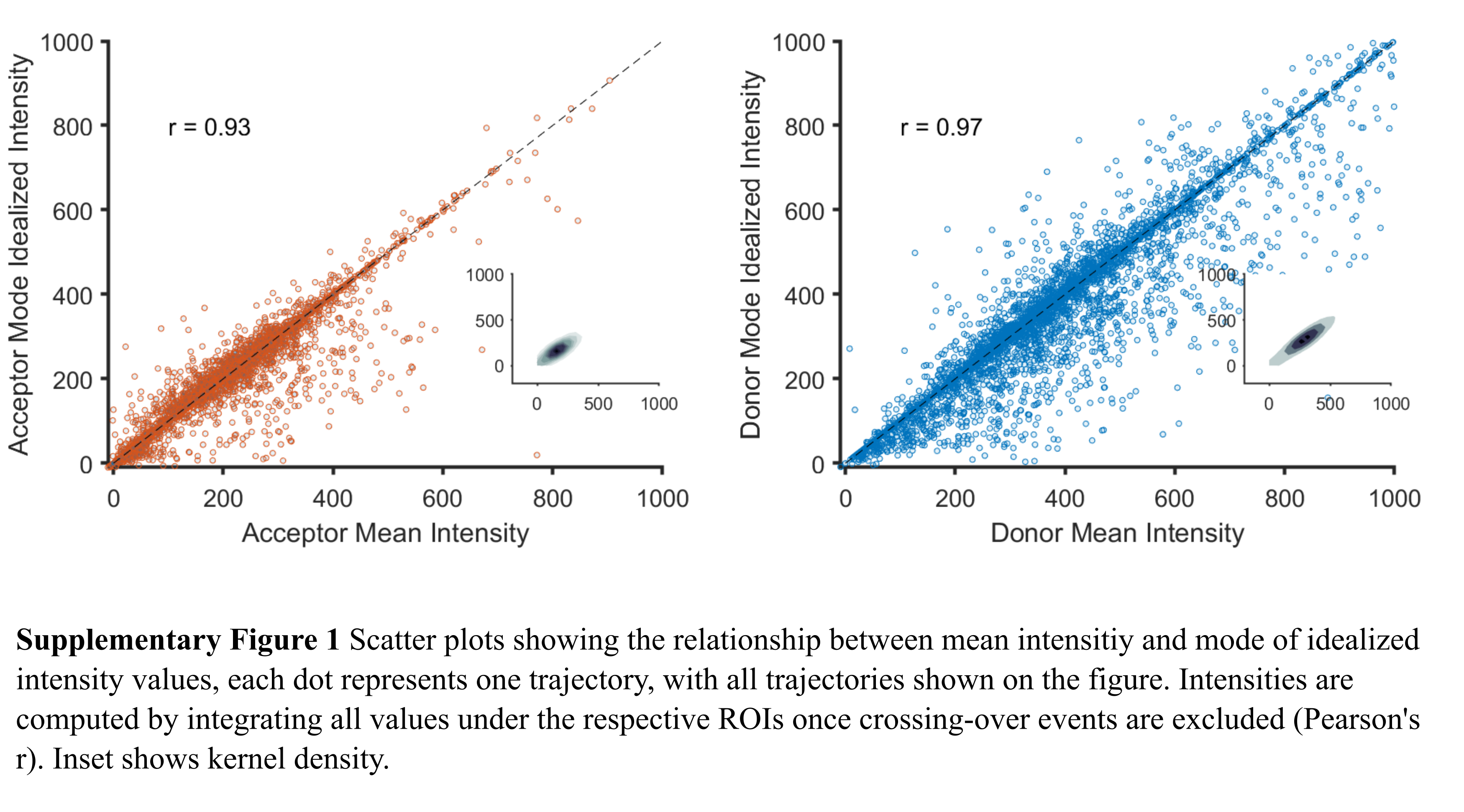

Supplement: Supplementary file 1 [file Figure5.TIFF]
